# Intracellular Localization and Cellular Factors Interaction of HTLV-1 and HTLV-2 Tax Proteins: Similarities and Functional Differences

**DOI:** 10.3390/v3050541

**Published:** 2011-05-09

**Authors:** Umberto Bertazzoni, Marco Turci, Francesca Avesani, Gianfranco Di Gennaro, Carlo Bidoia, Maria Grazia Romanelli

**Affiliations:** 1 Department of Life and Reproduction Sciences, University of Verona, Strada le Grazie 8, 37134, Verona, Italy; E-Mails: marco.turci@univr.it (M.T.); francesca.avesani@gmail.com (F.A.); gianfranco.digennato@univr.it (G.D.G.); 2 Centre for Research in Infectious Diseases, University College Dublin, Belfield, Dublin 4, Ireland; E-Mail: carlo.bidoia@ucd.ie (C.B.)

**Keywords:** HTLV, Tax-1, Tax-2, signal transduction, cellular localization, post-translational modifications, NF-κB, PDZ binding motif

## Abstract

Human T-lymphotropic viruses type 1 (HTLV-1) and type 2 (HTLV-2) present very similar genomic structures but HTLV-1 is more pathogenic than HTLV-2. Is this difference due to their transactivating Tax proteins, Tax-1 and Tax-2, which are responsible for viral and cellular gene activation? Do Tax-1 and Tax-2 differ in their cellular localization and in their interaction pattern with cellular factors? In this review, we summarize Tax-1 and Tax-2 structural and phenotypic properties, their interaction with factors involved in signal transduction and their localization-related behavior within the cell. Special attention will be given to the distinctions between Tax-1 and Tax-2 that likely play an important role in their transactivation activity.

## 1. Introduction

Human T-lymphotropic viruses type 1 (HTLV-1) and type 2 (HTLV-2) are closely related human retroviruses. HTLV-1 is tropic for CD4^+^ and HTLV-2 preferentially infects CD8^+^ T-cells [1]. HTLV-1 is the etiologic agent of adult T-cell leukemia/lymphoma (ATLL) and of HTLV-1-associated myelopathy/tropical spastic paraparesis (HAM/TSP) [2]. As yet, HTLV-2 is not known to have a precise pathologic role, and is not associated with any malignancies but only with rare cases of subacute myelopathy resembling HAM/TSP [3]. In addition to *gag*, *pol*, and *env* genes, HTLV-1 and HTLV-2 encode *tax/rex* and several accessory genes from the pX ORF located in the 3′ region of the genome [1,4]. The minus strands of HTLV-1 and HTLV-2 also code for proteins involved in cell proliferation, HBZ (HTLV-1 B-Zip protein) and APH-2 (HTLV-2 encoded antisense protein) [5,6].

HTLV-1 and HTLV-2 infectivity and transformation capacity appears to differ from that exerted by other animal retroviruses in that tumor development is not mediated by the presence of a viral oncogene or by the activation of a cellular proto-oncogene. Since HTLV is transmitted essentially through cell to cell contact and both viruses have the capability of inducing T-cell transformation [7,8], the ability of the oncogenic Tax proteins of HTLV-1 (Tax-1) and HTLV-2 (Tax-2) to induce cell proliferation becomes essential. In fact, Tax-1 plays a central role in triggering transforming activity, acting as a potent transcriptional activator of both viral and cellular genes [9]. More generally this molecule displays pleiotropic activities through the interaction with cellular transcriptional factors and signaling pathways which have been intensively studied [2,10]. This variety of effects concurs to the induction of neoplastic transformation by activating the expression of cytokines responsible for T cell proliferation [11], by abrogating DNA repair [12], thus increasing mutation load and DNA damage, and by suppressing apoptosis [13]. Recent studies have shown that Tax-1 transgenic mice develop T cell lymphomas, demonstrating that Tax is capable by itself of inducing an oncogenic effect *in vivo* [14,15]. Although the etiologic difference in pathogenic properties of HTLV-1 and HTLV-2 still remains unclear, it has been suggested that it could be attributed to the differential structure and activities of their transactivating Tax proteins. An impressive body of evidence has already been obtained suggesting that Tax-1 is involved in survival of HTLV-1 infected T-cells, in cell cycle progression and genome instability [10,12,16–19]. In this review, we will not discuss all different facets of Tax-1 effects, rather we will focus on common features and functional differences of Tax-1 and Tax-2 for which sufficient information has so far being accumulated, with the main view of updating recent reports and reviews [20].

Tax-1 and Tax-2 share an amino acid similarity of 85%. However, they present distinct phenotypic differences consistent with a more robust transformation capacity of Tax-1 in comparison to Tax-2 [1,21]. Compared to Tax-2, Tax-1 transactivates the viral LTR promoter with higher efficiency and has a unique capacity to induce micronuclei formation [22]. In addition, Tax-1 arrests the cell cycle of human CD34^+^ cells [23], suppresses hematopoiesis [13] and regulates gene expression through histone modifications [24,25]. Tax-2 was shown to be impaired for the inhibition of p53 functions [26] and to less efficiently transform rat embryo fibroblasts [21]. We have contributed to a report demonstrating that Tax-2 activation of HTLV-2 LTR is strongly inhibited by the MHC class II transactivator (CIITA) [27]. More recently, Tosi *et al.* [28] have shown that Tax-2 differs from Tax-1 since it is using CBP and p300 but not PCAF to enhance LTR transactivation. In a recent work, Li and Green [29] found that total viral mRNA expression was significantly higher for HTLV-1 than HTLV-2, which is consistent with a previous finding that HTLV-1 has stronger Tax transactivating activity than HTLV-2 [9]. Tax-1 expression, as compared to Tax-2, resulted in higher levels of proinflammatory cytokine expression in human astrocytomas [11].

## 2. Protein Structure

**Figure 1:**
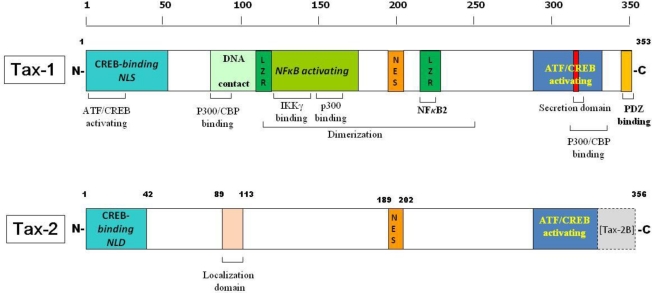
Structural and functional domains of Tax-1 and Tax-2. Specific domains, and the regions involved in transcriptional activation pathways are indicated.

The protein structure of Tax-1 and Tax-2 is presented as a scheme in Figure 1. Of the four serotype of HTLV-2, Tax-2 of subtype A and type B are the best characterized [30]. Tax-2A has 331 amino acid (aa) residues, whereas Tax-2B presents a 25 aa C-terminal extension [31,32]. The Tax-2 structure depicted in Figure 1 is that of Tax-2B with 356 aa, being very close to that of Tax-1, which is 353 aa long. Tax-1 and Tax-2 have several common domains, even though the information for Tax-1 is by far more complete. The N-terminal region of Tax-1 protein contains a CREB-binding region, a zinc-finger domain, and binding domains required for interaction with proteasomal subunits, transcriptional coactivators, and proteins involved in transcription, cell cycle progression, and in cell signaling regulations [1]. The first 60 aa of Tax-1 also contains a nuclear localization signal (NLS) [33,34], whereas we have mapped a nuclear localization determinant (NLD) in the first 42 aa of Tax-2 [35]. Furthermore, in Tax-2, an additional localization domain of about 10 aa at position 89–113 has been demonstrated to be responsible for the divergent cellular localization as compared to Tax-1 [36].

The central portion of Tax-1 contains two leucine zipper-like motifs at aa position 116–145 and 213–248 [17], necessary for interacting with DNA, as well as for protein dimerization [37,38].

This region also presents binding domains for proteins involved in histone methylation, cell cycle progression and in cell signaling transduction [17,34,39–42]. The central region of Tax-1 is also directly involved in NF-κB activation [17,43–47] association and p300 binding. The Tax-1 region encompassing aa 225–232, overlapping with the leucine zipper like region (LZR), is responsible for Tax1-mediated p100 processing and p52 nuclear translocation [48,49]. This sequence is not present within Tax-2, so that Tax-2 is unable to interact and process p100 [48,50]. Thus a Tax-2 LZR region involved in NF-KB2 activation has not been characterized. Another domain, which is common to the two proteins, is the nuclear export sequence (NES) at amino acid position 189–202 [43,51]. The C-terminal region is characterized by the presence in both Tax-1 and Tax-2 of an ATF/CREB activating domain, essential for transactivation of the CREB/ATF and for NF-κB/Rel signaling pathways [52].

Secretory signals are present at the C-terminal part of Tax-1 that confers to the protein the capability to egress from the endoplasmic reticulum and to migrate to the Golgi apparatus [53,54].

The presence of a class I PDZ binding motif (PBM) at the C-terminal end of Tax-1 and not in Tax-2 is of particular interest. It allows Tax-1 but not Tax-2B to interact with many cellular factors, mostly of them being scaffolding proteins (reviewed in [20]).

## 3. Tax-1 and Tax-2 Interaction with Factors Involved in Signal Transduction

Protein-protein interactions represent a relevant issue for deciphering Tax protein functions and its capability to perturb the regulation of cellular pathways. Tax-1 interacts with several factors that are components of the cell signaling system, that control activities strictly linked to cell transformation, including proliferation, intracellular protein distribution, cell migration and virological synapses [10,19,55,56]. Representative Tax-interacting cellular proteins together with their corresponding signaling pathways (G proteins, MAPK, JNK, AP1, TGFβ, and NF-κB signaling) are shown in Table 1 [47,57–65]. Whereas more than 100 proteins have been identified that interact with Tax-1 [17], only a restricted number of Tax-2 interactions are known and the greater part of them are involved in the NF-κB pathway. Therefore, to describe and compare Tax-1 and Tax-2B involvement in signal transduction, we will focus mainly to Tax-protein interaction with NF-κB factors.

**Figure 2:**
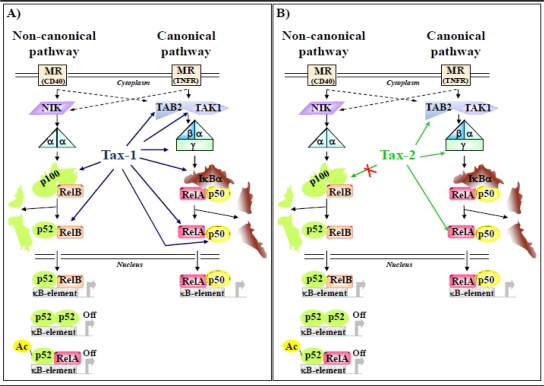
Tax-1 and Tax-2 interaction with NF-κB pathway. Schematic representation of the main steps in NF-κB pathaway. Non-canonical NF-κB activation involves the action of NF-κB inducing kinase (NIK) and IKKα subunit (α) and results in the C-terminal proteosomal degradation of p100 in p52 and in the nuclear translocation of NF-κB dimers containing p52. In the nucleus, the p52/RelB dimers induce gene expression, whereas p52 homodimers or acetylated p52/RelA heterodimers turn off gene transcription. Canonical NF-κB activation triggers a phosphorylation cascade involving kinases such as TAK1, adaptor proteins such as TAB2, and the IKK complex (here schematized as α, β and γ), and results in proteosomal degradation of cytoplasmic inhibitors (IκB) and in nuclear translocation of NF-κB dimers (mainly RelA/p50). In the nucleus, the RelA/p50 dimers induce gene expression for cytokines, enzymes and adhesion molecules. (**A**) Blue arrows identify Tax-1 interactions with NF-κB pathway factors; (**B**) Green arrows identify Tax-2 interactions or absence of interaction (red X) with NF-κB pathway factors.

The activation of NF-κB pathway is essential for Tax-mediated transformation of human T-cell lines by HTLV-1 and HTLV-2. Both Tax-1 and Tax-2 activate gene expression via NF-κB [70–72]. Of the two NF-κB-signaling pathways that mediate the transcriptional activation, the so-called canonical and non canonical, Tax-1 has been shown to deregulate both pathways by interacting with and activating several factors, including RelA and IκB kinase complex (e.g., IKKα, IKKβ, and NEMO/IKKγ) of the canonical signaling cascade [17], as well as p100, precursor of the non-canonical pathway (Figure 2A). The role of RelA in Tax-1-mediated activation of NF-κB has been analyzed in several published works. It has been shown that Tax-1 interacts with the Rel homology domain of RelA [73] and co-localizes with the NF-κB subunit RelA, the splicing factors Sm and SC-35 and the large subunit of RNA polymerase II in nuclear structures called nuclear bodies. [74–76]. Tax-1 interaction has been reported with TAK1-binding-protein 2 (TAB2) [77]. The expression of TAB2 increases in HTLV-1-transformed cells [77]. Tax-1 is present in the Golgi-associated lipid raft microdomains and directs IKK complex and Tak1 translocation to lipid raft through selective interaction with NEMO/IKKγ [71]. Compared to Tax-1, Tax-2 interacts with NEMO/IKKγ and the related proteins NRP/Optineurin [36,78] but does not recognize p100 [48] (Figure 2B). Moreover, like Tax-1, Tax-2 can associate to lipid rafts, but does not possess the ability to direct the lipid raft translocation of IKK [71]. Recently, we have provided evidence that Tax-2B, like Tax-1, is capable of interacting with additional factors of the NF-κB pathway, namely RelA and TAB2 [70], which do not associate with each other [79]. We have also shown that, in different cell types, both Tax-1 and Tax-2B relocalize RelA to cytoplasmic dotted structures which include TAB2, while in cells that do not express Tax proteins, overexpressed TAB2 does not co-localize with the diffuse cytoplasmic distributed RelA [70]. In addition, we showed that co-expression of RelA or TAB2 with Tax-1 or Tax-2B, synergistically enhanced NF-κB activity. Although it is still not clear the mechanism by which Tax proteins hijack NF-κB factors in the cytoplasm, it is plausible that both Tax proteins endow new properties to cellular proteins interacting with them. A similar property has been attributed to Tax-induced novel interaction between RelA and p53 that results in the deregulation of p53 transcriptional activity [80].

## 4. Post-Translational Modifications: Phosphorylation, Ubiquitination, Sumoylation and Acetylation

### 4.1. Tax Phosphorylation and its PDZ Binding Motif (PBM)

So far, six Tax-1 residues were identified as phosphorylation targets: Thr-48, Thr-184, Thr-215, Ser-300, Ser-301 and Ser-336 [81,82]. Serines at position 300 or 301 are essential for gene transcription and their phosphorylation is required in order to activate Tax-1 [81] and to consequently allow post-translational modifications such as ubiquitination, sumoylation and acetylation [83]. Phosphomimicking mutation at Thr-215 resulted in Tax-1 inhibition and mutations at Thr-48 impaired activation of NF-κB pathway, whereas mutations at Thr-184 and Ser-336 were found to be silent.

Recently, Jeong *et al.* [84] provided indirect evidence that Tax-1 phosphorylation at Ser-160 stabilizes Tax-1. The interaction of Tax-1 phosphoSerine-Proline motif with the prolyl isomerase Pin1 inhibited its ubiquitination and its lysosomal degradation. Ser-160 and Pro-161 were already identified as essential residues for the activity of Tax-1 by mutational analysis [85,86].

The phosphorylation state of Tax-2B is still unknown, even though it presents 85% homology to Tax-1 for the sequences surrounding the aforementioned phosphorylated residues, which are conserved with the exception of Ser-336. We have recently found that CK2 phosphorylates Tax-1 Ser-336, Ser-344 and Thr-351 *in vitro* [87]. Compared to Tax-1, Tax-2 was found not to be phosphorylated by CK2 *in vitro*.

Tax-1 C-terminus, which contains the class I PDZ binding motif (PBM, X-S/T-X-V, where X represents any aa) is completely different from that of Tax-2B. Thus it allows Tax-1, but not Tax-2B, to interact with many cellular factors, mostly scaffolding proteins, such as human disc large (hDlg), human homolog Scrib (hScrib), membrane-associated guanylate kinase-3 (MAGI-3), pro-IL16 and Erbin (reviewed in [20]). Disruption of hDlg or hScrib activity by Tax-1 through PDZ interaction revealed their function as tumor suppressors [88,89]. Tax-1 PBM phosphorylation could modulate PDZ-mediated interactions [87]. Interestingly, removal or loss-of-function mutations of PBM motif did not affect Tax-1 activation properties, but impeded the interaction with the tumor suppressor hDlg [87,90].

Recently, other PDZ containing proteins that interact with Tax-1 PBM were reported [91]. Of particular interest, these authors found that syntenin-2 interacts with Tax-1 by its PDZ domain and co-localizes with Tax-related nuclear bodies, but not with Tax-2. It is known that syntenin-2 is physiologically targeted to nuclear speckles through its strong PDZ interaction with phosphatidylinositol 4,5-bisphosphate (PIP2) and its depletion by RNA interference impairs cell viability and reduces the rate of cell division [92]. Syntenins are also involved in activation of NF-κB after Src kinase activation [93] These data suggest a new important role for Tax-1 PBM in recruiting syntenin-2 in the Tax-related nuclear bodies, and promoting its function.

### 4.2. Tax Ubiquitination and Sumoylation

Ubiquitination and sumoylation have been shown to play a critical role in the cellular localization, function and protein-protein interactions of both Tax-1 and Tax-2 [70,72,94–96].

**Figure 3:**
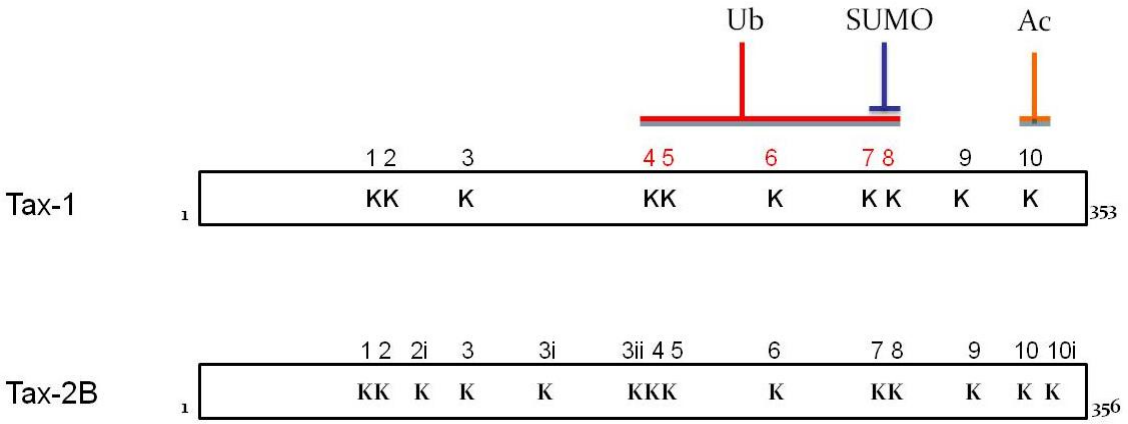
Comparison of Tax-1 and Tax-2 lysine content and modifications. Tax-1 lysines numbered 4, 5, 6, 7 and 8 have been proven to be the major sites for ubiquitination (Ub), while sumoylation occurs exclusively on the lysines 7 and 8 (SUMO). Acetylation occurs on lysine 10 (Ac). Lysines of Tax-2B from K1 to K10i are indicated. Lysine residues involved in Tax-2B modifications have not yet been precisely identified.

Five of ten lysine residues contained in Tax-1 sequence were found to be the major targets for these modifications (see Figure 3). Lys189 (K4), Lys197 (K5), Lys263 (K6), Lys280 (K7) and Lys284 (K8), all contained within Tax-1 C-terminal region, strongly contribute to the ubiquitination of the protein, while sumoylation takes place on Lys280 (K7) and Lys284 (K8) [75,76].

The role of ubiquitination and sumoylation in Tax-1 transactivating activity is clearly different. Tax-1 ubiquitination is involved in the activation of NF-κB pathway through the binding with key factors such as IKKγ and TAK-1 binding protein 2 (TAB2), with the subsequent RelA nuclear translocation [97,98].

Whether the poly-ubiquitination targets Tax to the proteasome or enhances its activity is still not clear, although Yan *et al.* [99] demonstrated that poly-ubiquitination of K63 stabilizes Tax-1 and this form is predominant in the cytoplasm. They also found that poly-ubiquitination of K48 is predominant in the nucleus and constitutively targets Tax-1 for proteasomal degradation. Those authors demonstrated that Tax-1 PBM interaction with PDLIM2 (PDZ and LIM domain protein 2, an ubiquitin E3 ligase containing a PDZ domain) directs cytoplasmic and nuclear Tax-1 to the nuclear matrix for ubiquitination-mediated degradation [99,100].

Other cellular factors have been demonstrated to be important in the post-translational regulation of Tax, such as peptidyl prolyl isomerase (Pin1) [84]. Pin1 prolongs the Tax-1 protein half-life by a mechanism that involves suppression of the ubiquitination and subsequent lysosomal degradation of Tax. Phosphorylated Tax-1 Ser160-Pro motif is recognized by Pin1 at the mitotic phase [84].

On the other hand, Tax-1 sumoylation is required to recruit RelA and IKKγ in Tax-1-related nuclear bodies, where Tax-driven transcription is promoted [74,76,94,101].

These data suggest that Tax-1 ubiquitination is a key event for controlling Tax-1 cytoplasmic/nuclear functions, whereas Tax-1 sumoylation is essential for its nuclear activity. Nonetheless, the predominance of ubiquitinated forms of Tax in the cytoplasm and of sumoylated forms in nuclear bodies is not a static event and can be affected by various signals. For example, it has been hypothesized that DNA damage regulates post-translational modifications of Tax and, consequently, its protein-protein interaction and localization [102].

**Figure 4:**
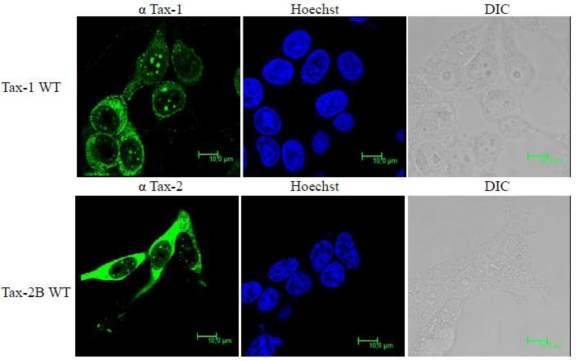
Comparison of Tax-1 and Tax-2 subcellular localization. Tax-1 and Tax-2B subcellular localization in 293T cells 24 h after transfection with vectors expressing both proteins. The cells were fixed and stained by immunofluorescence with anti-Tax-1 or anti-Tax-2B and analyzed by laser scanning confocal microscopy. Nuclear stain was performed using Hoechst stain.

Much less information has so far been obtained regarding Tax-2 post-translational modifications. We have recently demonstrated that Tax-2B is ubiquitinated and sumoylated similarly to Tax-1, even though with lower levels of mono-sumoylated, di-sumoylated and tri-sumoylated forms [72]. At variance from Tax-1, Tax-2 contains four additional lysine residues. The lysines involved in the modulation of Tax-1 activity are clearly defined, whereas the information for Tax-2B is still open (Figure 4).

### 4.3. Tax Acetylation

Tax-1 acetylation is known to be crucial for activating its functional properties, together with other post-translational modifications [83]. In particular, Ser-300/301 phosphorylation is a prerequisite for Tax-1 acetylation promoting its presence in the nucleus. Sumoylation is also necessary for the formation of Tax nuclear bodies where acetylation of the Tax-1 is performed by the transcriptional coactivator p300 [83].

Tax-1 acetylation results in the promotion of ATF/CREB and NF-κB pathways, probably by the activation of promoters integrated in the chromatin [83]. Tax-1 K10 (Lys346) (Figure 3), has been described as the only target of acetylation. Even though Tax-2 acetylation has not yet been studied in detail, evidence of its acetylation has already been obtained [83].

## 5. Cellular Localization of Tax Proteins

The pleiotropic activity of Tax proteins is characterized by specific interactions with cellular factors in the nucleus and in the cytoplasm. Consequently, there are specific mechanisms that modulate the cellular trafficking of these proteins. A number of studies have reported nucleo-cytoplasmic shuttling of Tax-1 and functional effects of subcellular localization of Tax, although the regulatory mechanisms of its intracellular distribution remain to be clarified [53]. In a recent work, the role of a histone methyltransferase (HMTase) SMYD3 in the regulation of Tax-1 nucleo-cytoplasmic shuttling has been reported [103]. They showed that the interaction between Tax and SMYD3 regulates the sub-cellular localization of Tax, as a consequence of SMYD3 shuttling and modifying Tax-mediated NF-κB activation.

Nuclear import-export of Tax-1 is both carrier and energy independent and relies on the interaction between Tax-1 and the p62 nucleoporin. This interaction is mediated by the amino terminal zinc-finger motif of Tax-1. Several mutations within this motif abolishes Tax1 interaction with p62 and nuclear import [104].

Initial studies on the intracellular distribution of Tax-1 indicated that it was localized essentially in the nucleus [22,33,34,86,105]. Using different cell lines, Tax-1 was then found to be prevalently present in subnuclear domains, which overlap with structures previously identified as interchromatin granules or spliceosomal speckled structures. These structures correspond to a subset of nuclear transcriptional hot spots. The inclusion of the two subunits of NF-κB, p50 and RelA, and the presence of the mRNA from a gene specifically activated by Tax through NF-κB binding, suggested that these unique nuclear structures, called nuclear bodies, participate in Tax-mediated activation of gene expression via the NF-κB pathway [74].

However, other studies have reported Tax-1 cytoplasmic localization in a number of cell lines including Tax-1-transfected and HTLV-1-infected cells [106–108]. In the cytoplasm, Tax-1 targets I-κBα and I-κBβ for phosphorylation, ubiquitination, and proteasome-mediated degradation, promoting the nuclear translocation of NF-κB/Rel proteins and the transcription induction of many cellular genes [109]. More specifically, cytoplasmic Tax-1 was demonstrated to localize within organelles associated with cellular secretory pathways, including the endoplasmic reticulum and the Golgi complex. [43].

Tax-1 is accumulated in close association with the Microtubule Organizing Center (MTOC) and is found in the cell-cell contact regions that organize virological synapses formed between an infected donor cell and a target cell [110,111]. In PBMCs derived from HTLV-1 infected patients, the fraction of Tax-1 present at the MTOC is closely associated with the *cis*-Golgi compartment [112].

Tax-2 intracellular localization has been studied by several authors and it was initially reported to be predominantly localized in the cytoplasm [30,35,36,51,113] with no clear evidence of localization within the nuclear bodies. However, using novel systems to compare concomitantly Tax-1 and Tax-2, we have demonstrated that Tax-2B is also present in nuclear bodies [72]. The comparison between Tax-1 and Tax-2 intracellular distribution is exemplified in Figure 4, obtained by using a new specific antibody against Tax-2 [114]). It is evident that the proteins are localized in both nuclear and cytoplasmic compartments, but Tax-2 is more markedly distributed in the cytoplasm. Localization within the nuclear bodies is also evident for both proteins, though those produced by Tax-1 are more prominent and copious.

From the functional standpoint, it is important to note that the Tax proteins are targeted to native TAB2-containing cytoplasmic structures, which are reorganized to include RelA and calreticulin following expression of Tax proteins [70]. In addition to TAB2 and RelA, other significant factors such as TAX1BP1 and IKKγ have been recently detected in the dotted cytoplasmic structures containing Tax [70].

## 6. Concluding Remarks

Much attention has been dedicated to the understanding of the functional and structural properties of Tax proteins and in particular of Tax-1. All the different facets of its structural domains, its interaction with cellular factors and corresponding deregulation of transduction pathways, its intracellular distribution as well as its post-translational modifications, have been critically and minutely dissected (for a recent review, see [20]). Much less is known for Tax-2, even though a more advanced comparative analysis between the two proteins could be of particular interest in reaching a better understanding of the differential pathogenic potential between HTLV-1 and HTLV-2.

In Table 2 we have summarized the main functional and structural differences between Tax-1 and Tax-2. Besides the notable differences in transformation and expression capacities, there are two basic structural features that differentiate Tax-1 from Tax-2: the presence only in Tax-1 of a NF-κB2 domain and a PDZ binding motif, responsible for the binding to p100 and several PDZ-containing proteins, respectively [49].

**Figure 5:**
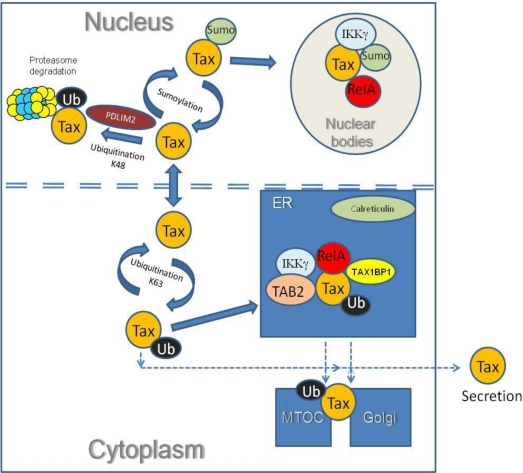
A model for Tax trafficking in the cell. The figure shows the intracellular distribution of Tax-1 after ubiquitination and sumoylation post-translation modifications. Tax-1 colocalizes in calreticulin stained dotted structures including TAB2, RelA, IKKγ and TAX1BP1 in endoplasmic reticulum (ER). In the cytoplasm, ubiquitinated Tax activates IKK signaling, resulting in the translocation of the NF-κB RelA factor to the nucleus. The de-ubiquitinated Tax migrates to the nucleus where it is sumoylated and assembled in nuclear bodies that include the RelA and IKKγ. Tax-1 poly-ubiquitination on K48 targets Tax-1 for proteasomal degradation, after interacting with PDLIM2. The scheme also proposes the trafficking of Tax to the Golgi, the Microtubule Organizing Center (MTOC) and outside the cell (secretion).

Central to the question of subcellular localization is the issue of post-translational modifications of both proteins. The recent findings that Tax-2 is also modified by ubiquitination and sumoylation [72] and that both proteins present similar interaction capacity with specific cellular factors [70] have shed light on the existence of a common intracellular distribution and interactome, as depicted in the scheme of Figure 5. Poly-Ubiquitination (ubiquitination on Lys63) is a crucial step in maintaining Tax-1 within the cytoplasm [99], followed by its transport into the ER together with RelA, IKKγ, TAB2 and TAX1BP1 and poly-ubiquitination on Lys48, promoted by PDLIM2, leads Tax-1 to nuclear matrix for proteasomal degradation [99,100].

Possible other ways leading to the Golgi, to the MTOC and to secretion, still await further confirmation, especially for Tax-2. The sumoylation process is key to nuclear entry and maintenance, and a prerequisite for Tax localization within the Tax-related nuclear bodies. This post-translational modification appears to represent a subtle difference between the two proteins, inasmuch as only poly-sumoylated forms are clearly evident for Tax-2 [72]. Furthermore, no evidence for Tax-2 CK2 phosphorylation has so far been obtained [87].

More insights and efforts are therefore needed to single out possible differences between Tax-1 and Tax-2 in the post-translational processing and in obtaining a more precise identification of the amino acid residues directly involved in the modifications of the two proteins.

## Figures and Tables

**Table 1:** Cell signaling factors interacting with Tax-1.

**Pathways**	**Factors**	Reference
G proteins	Gβ subunit	[47,66]
	RhoGTPases	

MAPKs	MEKK1	[59,61]
	TAK1	

JNK	GPS2	[61]

AP1	p85α	[67]

TGFβ	Smad2	[64]
	Smad3	[64]
	Smad4	[64]

NF-KB	IKKα	[65]
	IKKβ	[65]
	IKKγ	[65]
	IκBα	[68]
	IκBγ	[69]

**Table 2:** Summary of main functional and structural differences between Tax-1 and Tax-2.

	**Tax-1**	**Tax-2^a^**	**Reference**
Transactivating activity	Higher	Lower	[113]
Transformation capacity	Higher	Lower	[21]
Micronuclei formation	+	−	[113]
Cell cycle arrest	+	−	[23]
Hematopoiesis suppression	+	−	[13]
Reduction of histone gene expression	+	−	[24,25]
Inhibition of p53 functions	Higher	Lower	[26,115] [80]
Total viral mRNA expression	Higher	Lower	[29]
Proinflammatory cytokine expression	Higher	Lower	[11]
Presence of PDZ motif	+	−	[1]
Interaction with PDZ binding proteins	+	−	[20]
Presence of NF-κB2 protein domain	+	−	[49]
Interaction with p100	+	−	[49]
Preferential cellular localization	Nucleus	Cytoplasm	[72]
NF-κB transactivation (lipid raft translocation of IKK)	+	−	[70]
*In vitro* CK2 phosphorylation	+	−	[87]
Oligo-sumoylation	+	−	[72]
Nuclear bodies	Larger	Smaller	[72]

a The properties of Tax-2 include both the results of Tax-2A and Tax-2B reported in the literature.
